# *Ex Vivo* Estimation of Photoacoustic Imaging for Detecting Thyroid Microcalcifications

**DOI:** 10.1371/journal.pone.0113358

**Published:** 2014-11-21

**Authors:** Jeeun Kang, Woong Youn Chung, Sang-Wook Kang, Hyeong Ju Kwon, Jaeheung Yoo, Eun-Kyung Kim, Jin Ho Chang, Tai-kyong Song, Sohee Lee, Jin Young Kwak

**Affiliations:** 1 Department of Electronics Engineering, Sogang University, Seoul, Korea; 2 Department of Surgery, Severance Hospital, Yonsei University College of Medicine, Seoul, Korea; 3 Department of Pathology, Severance Hospital, Yonsei University, College of Medicine, Seoul, Korea; 4 Yonsei University, College of Medicine, Seoul, Korea; 5 Department of Radiology, Severance Hospital, Research Institute of Radiological Science, Yonsei University College of Medicine, Seoul, Korea; 6 Interdisciplinary Program of Integrated Biotechnology, Sogang University, Seoul, South Korea; 7 Department of Surgery, The Catholic University of Korea, Seoul, South Korea; NIH, United States of America

## Abstract

**Background:**

The aim of this study was to evaluate the diagnostic utility of PAI at detecting thyroid microcalcifications at 700 nm laser wavelengths.

**Methods:**

This study included 36 resected samples in 18 patients. To evaluate the PA manifestation of microcalcifications in PAI, gray level histogram and co-occurrence matrix (COM) texture parameters were extracted from the 3 fixed ROI US and PA images, respectively, per sample. We compared the textural parameters obtained from specimen PAIs between samples with punctate microcalcifications on specimen radiography and those without microcalcifications.

**Results:**

On specimen US, the mean value (2748.4±862.5) of samples with microcalcifications on specimen radiography was higher than that (1961.9±780.2) of those without microcalcifications (*P* = 0.007). However, there were no significant differences in textural parameters obtained from specimen PAIs between samples with punctate microcalcifications on specimen radiography and those without when applying both the mean value of the three slices of thyroid specimens and the value of the thyroid specimen slice which had the highest value of the mean values in specimen US.

**Conclusion:**

PAI did not show significant PA contrast on thyroid microcalcifications indicating that the experimental setup and protocols should be enhanced, e.g., method of complete blood rejection from *ex vivo* specimens, the multi-wavelength spectroscopic PA imaging method which can solely extract the PA signal from microcalcifications even with high spectral interferences, or PA imaging with narrower slice thickness using 2-dimensional array transducer, etc.

## Introduction

High-frequency ultrasonography (US) is a highly sensitive diagnostic tool that detects thyroid calcifications as well as thyroid nodules. On US, hyperechoic foci with or without acoustic shadowing have been interpreted as calcifications with the sensitivity and specificity of US for diagnosing a malignancy reported to be 26.1–59.1% and 85.8–95.0%, respectively [1]. However, US has shown some drawbacks in the detection of thyroid microcalcifications which are related to thyroid malignancies [2]. In the field of breast cancer diagnosis, mammography has been widely used on lesions which are not visible on US to document breast calcifications related to breast cancers [3]. Although thyroid calcifications can only be seen through specimen radiography, there have been no preoperative clinical tools that detect thyroid calcifications like mammography does with breast calcifications until now [2].

Photoacoustic imaging (PAI) is a raising imaging modality that uses the different optical absorption properties of tissues and it provides high spatial and contrast resolutions [4], [5]. When pulsed laser light is emitted into targets, the targets absorb energy. Then, a transient temperature rise is converted to a pressure rise by thermoelastic expansion and subsequently photoacoustic signals are generated. The signals are received by US transducers and then imaged via several reconstructive techniques [6]–[8]. Since the absorption coefficients of oxygenated- and deoxygenated-hemoglobin in blood are higher than that of surrounding tissues and the optical absorption is different according to the type of molecule as well as tissue, PAI provides functional information about tissue oxygenation and vascularization [9]. Also, integrated US and PAI have been introduced in a variety of clinical fields such as diagnoses of ovarian cancer [10], [11], breast cancer [12], and inflammatory joint diseases [13], and the detection of lipid deposits [14] and breast microcalcifications [15].

Through previous *ex vivo* experiments, we have found that the optimal laser wavelength of PAI to detect breast microcalcifications which were not detected on US is in the 690–700 nm range [15]. Although thyroid glands have different tissue characteristics from breast tissue, we assume that microcalcifications have the same absorption coefficients regardless of tissue type. In this study, we evaluate the diagnostic utility of PAI at detecting thyroid microcalcifications at 700 nm laser wavelengths.

## Subjects and Methods

Human thyroid tissues were obtained following the approval of the Institutional Review Board (IRB) of Severance Hospital (Seoul, Korea). Informed written consent was obtained from all patients and the study was performed in accordance with the ethical guidelines of the Helsinki Declaration. The study period was from Dec 2012 to Mar 2014. All patients scheduled for thyroid cancer surgery at the surgery department of our hospital were evaluated for inclusion. For this study, specimen radiographs were immediately obtained after surgery. Specimen radiographs were evaluated in comparison to staging ultrasound (US) images by one radiologist who decided the study candidates. After reviewing the two images, two thyroid tissue samples (about 20×5×5 mm, length × width × depth) from different lobes were obtained from the specimens, with one containing compact punctate calcifications and the other not containing any calcifications. This sample collection did not interfere with the pathologic diagnosis process. To verify calcifications, specimen radiographs were also obtained at resected thyroid tissues. Initially, 54 samples from 27 patients were obtained for this study. Eighteen samples in 9 patients were excluded because they had blood coagulations on the samples, which were formed after the resection and would possibly introduce a big bias on the result. Therefore, of the initial 54 samples, 36 resected samples from 18 patients were included as the final subjects of this study. The selected specimens were secondly washed out by being steeped in saline solution for 6 hours.

### Imaging by ultrasonography and specimen radiography [2]

Thyroid US was performed with a 5–12-MHz linear-array transducer (iU22; Philips Medical Systems). Compound imaging was performed in all cases. Real-time ultrasonography was performed by one of 7 radiologists specializing in thyroid imaging. The scanning protocol in all patients included both transverse and longitudinal real-time imaging of the thyroid, with the use of a picture archiving and communications system to review all patient data. Microcalcifications revealed by US were defined as hyperechoic punctate foci with or without acoustic shadowing, excluding dense round calcifications or condensed colloids showing a comet tail artifact.

After surgery, specimen radiographs were obtained using the Lorad/Hologic Selenia FFDM system (Lorad/Hologic, Danbury, Connecticut, USA), which was a dedicated mammography unit during the study period. The system, based on a detector with amorphous selenium, used a direct-capture, 70-µm pixel device and yielded a 2560×3328 matrix image with an 18×24-cm paddle. The system was set to allocate 16-bit images and store them at 12 bits. Routine views of thyroid specimens were obtained (focal spot size 0.3 mm). These images were displayed on a pair of high-resolution, 5-megapixel LCD monitors (MFGD 5621HD; Barco, Buluth, Georgia, USA) that were part of the review workstation (Selenia Softcopy WorkstationTM; Lorad/Hologic) with soft-copy reading software (MeVis BreastCare; MeVis Medical Solutions, Bremen, Germany).

### *Ex vivo* experimental setup for photoacoustic imaging

**Figure 1:**
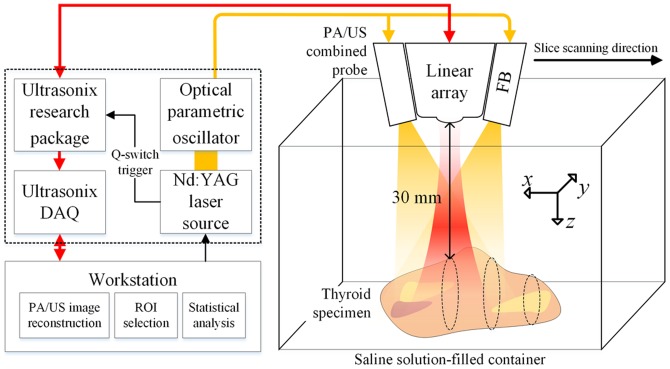
System configuration for the *ex vivo* experiments with a 1-dimensional photoacoustic/ultrasound combined probe. Note that the FB stands for the optical fiber bundle which delivers the laser energy to the thyroid specimen and the *x*, *y*, and *z* direction indicate the elevation, lateral, and axial direction from the PA/US combined probe, respectively.

After acquiring X-ray specimen radiographs, the *ex vivo* experiments were conducted with the PA/US imaging system shown in Figure 1. For the experiments, the respective thyroid specimens were set on a scatter-free gel pad (Aquaflex, Parker Lab, Inc., Farfield, NJ, USA), which was immersed in a 0.9% saline-filled container. The temperature in the container was stably maintained at 24°C. For the experiments, radio-frequency (RF) echo data for USI and PAI were captured with a commercial US scanner equipped with a SonixTouch research package (Ultrasonix Corp., Vancouver, BC, Canada) and a 5–14-MHz linear array transducer connected to a 128-channel SonixDAQ parallel system. The Q-switch trigger of a Nd:YAG laser-pumped OPO system (Surelite III-10 and Surelite OPO Plus, Continuum Inc., Santa Clara, CA, USA) was generated whenever the laser pulse was excited with a 10 Hz rate and then it was sent to a US scanner. By referring the Q-switch trigger, the dedicated data acquisition system stored a scanline and a frame RF channel data for PAI and USI, respectively. The laser pulse duration was 7 ns and its wavelength was fixed at 700 nm by a software program in the workstation. The distance between the array transducer and thyroid specimen was fixed at 30 mm, which was the geometrical focusing point of a custom bifurcated optic fiber bundle (Fiberoptic Systems, Inc., Simi Valley, CA, USA). The energy density was maintained at 10 mJ/cm^2^, which is under the ANSI radiative safety standards [17]. The energy density was ascertained by averaging 500 sequential pulses with an energy meter and sensor (MAESTRO and QE25, Gentec Electro-Optics Inc., Quebec, QC, Canada). From the experimental setup of PAI, three PA image slices were acquired from the respective resected thyroid tissues, which seemed to contain the calcification-suspicious foci on the corresponding US image slices regardless of the existence of microcalcifications. Afterwards, for the selected slices, regions-of-interest (ROIs) were chosen within an area the size of 0.5 cm and 0.5 cm in the axial and lateral directions, respectively. Note that the slices and ROIs were carefully chosen by a graduate researcher (K.J.) not to include the hyperreactive artifact regions in PA images, which are suspected to be from the partially-distributed blood remained despite of the 6-hour wash-out procedure with saline solution. Also, it should be noted that the selection of all the image slices and ROIs were conducted without specimen radiographs, thereby the statistical texture analysis parameters could be blindly computed for the respective thyroid specimens.

### Computation of texture analysis parameters

To evaluate the PA manifestation of microcalcifications in PAI, gray level histogram and co-occurrence matrix (COM) texture parameters [16] were extracted from the 3 fixed ROI US and PA images, respectively, per sample by using in-house software developed with Matlab R2010a (MathWorks, Natick, Massachusetts). Gray level histogram parameters consisted of mean, mean variance, skewness, kurtosis, and entropy, which presented a graphical distribution of the echogenic intensity values of pixels in the ROI. The COM parameters consisted of contrast, correlation, uniformity, homogeneity, and entropy, which described the joint probability of pixel pairs along all directions at different distances in the ROI. The co-occurrence matrix represented the spatial relationship of two neighbor pixels based on intensity level. First, ROI data were converted into P level images where P is a positive integer. P, the number of the level, has a relationship with SNR (Signal-to-Noise Ratio). Usually, a low level number corresponds to the robustness of algorithm to noise but it has limitations such as a lack of spatial information. In order to define the spatial relationship between two pixels, co-occurrence distance and direction should be defined in advance. Classification performance is related with co-occurrence distance and direction parameters because of the character of target texture. Therefore, to acquire optimal results, several co-occurrence matrices were calculated by adjusting the level number, co-occurrence distance and direction. In this study, ROI data were converted into 4, 6, 8, 10 level images. From these converted images, co-occurrence matrices were calculated in the orthogonal or diagonal direction and a co-occurrence matrix distance of 1 and 3. COM parameter values were generated from the co-occurrence matrix. Contrast, correlation, uniformity, homogeneity and entropy defined by Haralick et al were used as COM parameters [16].

### Specimen pathology

After conducting PAI, all resected specimens were fixed in 10 percent formalin and cut at 3-mm intervals. Surgical specimens were embedded in paraffin wax and stained with haematoxylin and eosin for histological examination. A pathologist, who did not have any prior information about the US and specimen radiographs, evaluated the presence of psammoma bodies (PBs) in the specimens.

### Statistical Analysis

To compare continuous variables, the independent two-sample *t*-test or the Mann-Whitney *U* test was used according to the normality from the Kolmogorov-Smirnov test. Continuous variables were presented as means ± standard deviation (SD) or as medians with interquartile range. Statistical significance was assumed when the *P* value was less than or equal to.05. All reported *P* values are two-sided. All analyses were performed using SAS statistical software (SAS system for Windows, version 9.1.3; SAS Institute, Cary, NC).

## Results

During the study period, there were 36 resected thyroid tissues. While 18 tissues contained compact punctate calcifications on specimen radiography, the other 18 tissues did not. Among the 18 resected samples with punctate microcalcifications on specimen radiography, 14 had PBs on pathology. In contrast, 5 samples of the tissues without punctate microcalcifications on specimen radiology had PBs on pathology.

**Figure 2:**
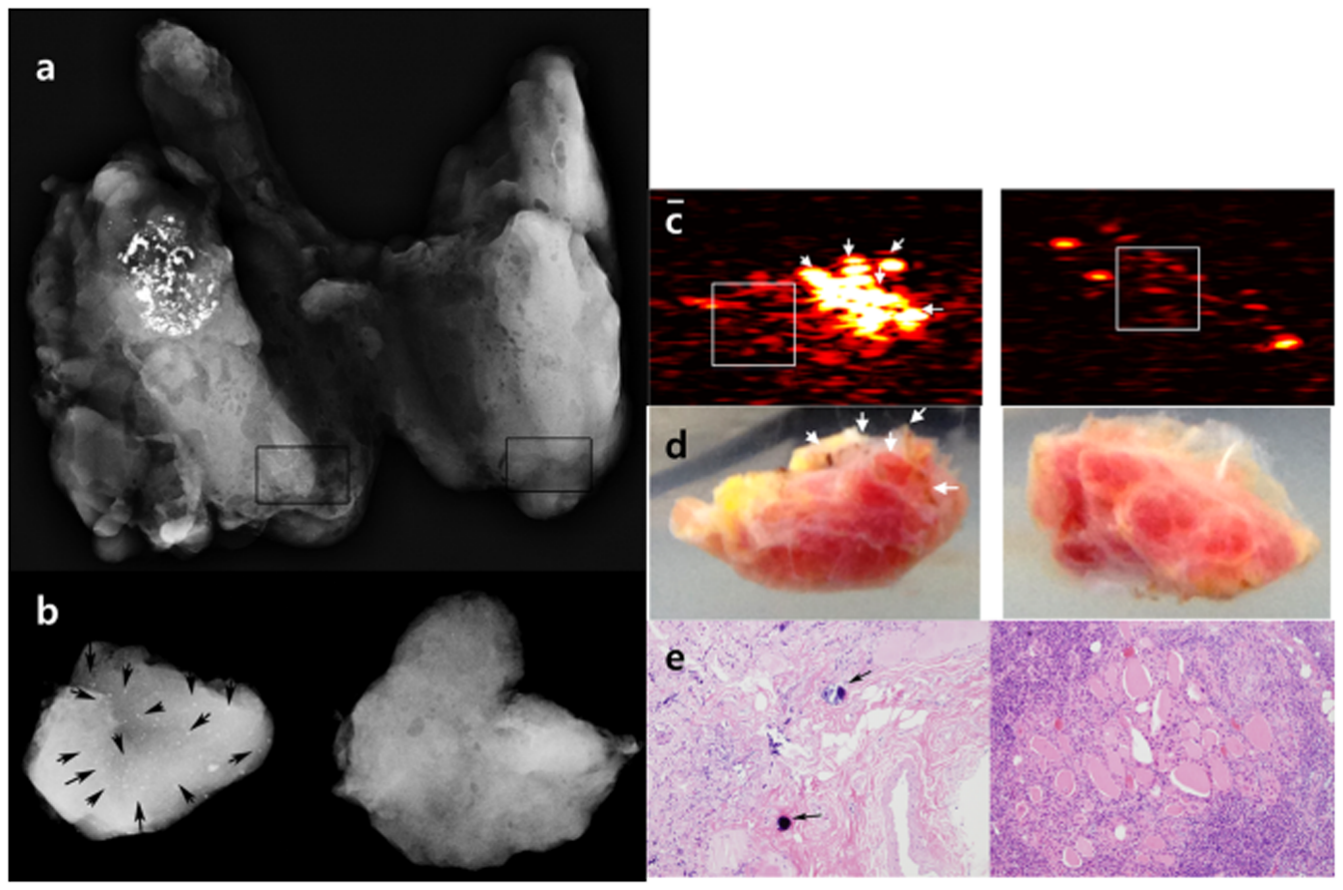
A 46-year-old male underwent total thyroidectomy due to thyroid cancer. A specimen radiograph (a) showed a mass with calcifications in the upper and mid pole of the right thyroid gland and punctate microcalcifications in the lower pole of the right thyroid gland which were not seen on US. There were no discernible calcifications in the left thyroid gland. To verify calcifications on PAI, two thyroid tissues from different lobes were obtained from thyroid specimens, with tissue from the right lobe containing compact punctate calcifications and tissue from the left lobe not containing any calcifications (two square boxes). The tissues were retrieved from the thyroid specimen without disturbing pathologic diagnosis. A specimen radiograph (b) showed punctate microcalcifications (arrows) acquired from the right thyroid gland and no calcifications acquired from the left thyroid gland. The texture analysis was conducted for the selected region-of-interests (ROIs) indicated by the white boxes in PA images (c) at 700-nm lasing wavelength. Note that the clustered hyperreactive PA signals suspected from the blood coagulations (white arrows) were excluded in the texture analysis, which can also be found on the corresponding photographs (d). In visual assessment, the ROI of the right resected tissue with microcalcifications contains more PA signals than that of the left resected tissue without microcalcifications. Correspondingly, the mean values of both US and PA images calculated from the right thyroid gland were higher than that from the left thyroid gland. There were psammoma bodies from a right resected tissue and no psammoma bodies (arrows) (H&E; X100) from a left resected tissue on pathology (e).

**Figure 3:**
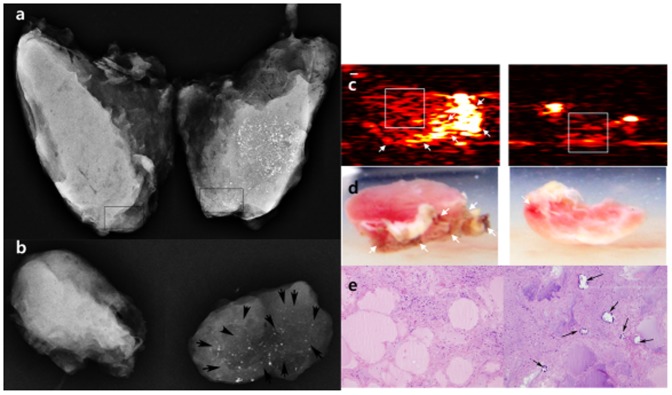
A 43-year-old male underwent total thyroidectomy due to thyroid cancer. A specimen radiograph (a) showed a mass with diffuse punctate calcifications in the left thyroid gland and no discernable calcifications in the right thyroid gland. To verify calcifications on photoacoustic imaging, two thyroid tissues from different lobes were obtained from thyroid specimens, with tissue from the left lobe containing compact punctate calcifications and tissue from the right lobe not containing any calcifications (two square boxes). The tissues were retrieved from the specimen without disturbing pathologic diagnosis. A specimen radiograph (b) showed punctate microcalcifications (arrows) acquired from the left thyroid gland and no calcifications acquired from the right thyroid gland. The texture analysis was conducted for the selected region-of-interests (ROIs) indicated by the white boxes in PA images (c) at 700-nm lasing wavelength. Note that the clustered hyperreactive PA signals suspected from the blood coagulations (white arrows) were excluded in the texture analysis, which can also be found on the corresponding photographs (d). In visual assessment of PA image, the ROI of the right resected tissue with microcalcifications contains more PA signals than that of the left resected tissue without microcalcifications. Although the mean value of samples from the left thyroid gland was higher than that of those from the right thyroid gland on specimen US, the mean value of samples from the right thyroid gland was higher than that of the left thyroid gland on both photoacoustic images. There were psammoma bodies from a left resected tissue and no psammoma bodies (arrows) (H&E; X100) from a right resected tissue on pathology (e).

On specimen US, the mean value (2748.4±862.5) of samples with microcalcifications on specimen radiography was higher than that (1961.9±780.2) of those without microcalcifications (*P* = 0.007). However, there were no significant differences in textural parameters obtained from specimen PAIs between samples with punctate microcalcifications on specimen radiography and those without when applying both the mean value of the three slices of thyroid specimens and the value of the thyroid specimen slice which had the highest value of the mean values in specimen US (Tables 1 and 2, Figures 2 and 3). When we compared textural parameters obtained from specimen PAIs between the samples with PBs on pathology and those without after applying the mean value of the three slices of thyroid specimens, there were also no significant differences between the tissues with and without PBs (Table 3).

**Table 1:** Comparison of textural parameters obtained from specimen photoacoustic images between tissues with punctate microcalcifications on specimen radiology and those without when applying the mean value of the three slices of thyroid specimens.

		Parameters	Mean ± SD or median (interquartile range)		*P* value
			Specimens without calcifications	Specimens with calcifications	
**Histogram**		Mean	39.964(24.296–49.04)	30.168(26.347–42.565)	0.3843
		Std*	25.636(14.914–32.226)	17.69(14.041–24.058)	0.1892
		Skewness	1.72(1.606–2.195)	1.571(1.177–1.938)	0.275
		Kurtosis	8.334(7.238–12.199)	7.396(5.162–9.322)	0.3346
		Entropy	6.075±0.63	5.917±0.646	0.4622
**Co-occurrence matrix**	Level†: 4 Distance‡: 1 Orthogonal§	Contrast	0.033(0.009–0.046)	0.013(0.006–0.043)	0.4572
		Correlation	0.903(0.859–0.932)	0.857(0.839–0.897)	0.0535
		Uniformity	0.756±0.175	0.801±0.205	0.4842
		Homogeneity	0.984(0.977–0.996)	0.994(0.978–0.997)	0.4572
		Entropy	0.514±0.345	0.423±0.415	0.4819
	Level†: 6 Distance‡: 1 Orthogonal§	Contrast	0.07(0.03–0.097)	0.035(0.025–0.101)	0.6016
		Correlation	0.904±0.04	0.894±0.039	0.452
		Uniformity	0.472(0.381–0.809)	0.695(0.418–0.759)	0.4964
		Homogeneity	0.965(0.952–0.985)	0.982(0.95–0.988)	0.6016
		Entropy	0.927±0.497	0.809±0.53	0.4967
	Level†: 8 Distance‡: 1 Orthogonal§	Contrast	0.094±0.052	0.085±0.054	0.6187
		Correlation	0.918±0.031	0.906±0.037	0.2818
		Uniformity	0.323(0.284–0.615)	0.488(0.292–0.593)	0.6016
		Homogeneity	0.953±0.026	0.958±0.027	0.6255
		Entropy	1.273±0.53	1.16±0.556	0.5368
	Level†: 10 Distance‡: 1 Orthogonal§	Contrast	0.124±0.061	0.113±0.063	0.6083
		Correlation	0.929±0.028	0.916±0.033	0.2137
		Uniformity	0.259(0.223–0.461)	0.372(0.242–0.456)	0.5166
		Homogeneity	0.939±0.03	0.944±0.031	0.6167
		Entropy	1.556±0.531	1.439±0.559	0.5234
	Level†: 10 Distance‡: 3 Orthogonal§	Contrast	0.4±0.216	0.357±0.231	0.5702
		Correlation	0.781±0.08	0.752±0.09	0.3243
		Uniformity	0.177(0.152–0.383)	0.299(0.159–0.392)	0.6016
		Homogeneity	0.845±0.07	0.856±0.073	0.6656
		Entropy	1.895±0.645	1.761±0.678	0.5478
	Level†: 10 Distance‡: 3 Diagonal§	Contrast	1.02(0.46–1.357)	0.602(0.408–0.992)	0.3346
		Correlation	0.53±0.096	0.49±0.096	0.2224
		Uniformity	0.139(0.113–0.337)	0.223(0.135–0.332)	0.4765
		Homogeneity	0.718(0.692–0.826)	0.791(0.715–0.825)	0.6239
		Entropy	2.133±0.706	1.995±0.75	0.5759

*Std, standard deviation.

† Level, number of level.

‡ Distance, co-occurrence distance (pixel).

§ Orthogonal and diagonal, co-occurrence direction with ± (1,0) and ± (1,1), respectively.

**Table 2:** Comparison of textural parameters obtained from specimen photoacoustic images between tissues with punctate microcalcifications on specimen radiology and those without when applying the value of a thyroid specimen slice which has the highest value of the mean values in the specimen ultrasound.

		Parameters	Mean ± SD or median (interquartile range)		*P* value
			Specimens without calcifications	Specimens with calcifications	
**Histogram**		Mean	35.869(25.633–42.321)	31.681(26.286–43.658)	0.937
		Std	23.08(14.291–28.493)	20.152(12.377–25.341)	0.4018
		Skewness	2.036±0.849	1.55±0.757	0.0787
		Kurtosis	10.348±6.106	7.106±4.312	0.0745
		Entropy	6.036±0.673	5.991±0.692	0.8455
**Co-occurrence matrix**	Level†: 4 Distance‡: 1 Orthogonal§	Contrast	0.022(0.009–0.039)	0.014(0.004–0.045)	0.7397
		Correlation	0.895±0.054	0.863±0.085	0.2196
		Uniformity	0.772(0.683–0.947)	0.87(0.668–0.976)	0.8618
		Homogeneity	0.989(0.98–0.995)	0.993(0.977–0.998)	0.7397
		Entropy	0.486±0.375	0.461±0.45	0.8565
	Level†: 6 Distance‡: 1 Orthogonal§	Contrast	0.057±0.041	0.06±0.047	0.849
		Correlation	0.911±0.041	0.894±0.057	0.3253
		Uniformity	0.589±0.238	0.595±0.242	0.9446
		Homogeneity	0.971±0.021	0.97±0.023	0.848
		Entropy	0.888±0.514	0.874±0.563	0.9423
	Level†: 8 Distance‡: 1 Orthogonal§	Contrast	0.088±0.053	0.092±0.059	0.8295
		Correlation	0.923±0.036	0.904±0.049	0.1954
		Uniformity	0.449±0.221	0.442±0.218	0.9267
		Homogeneity	0.956±0.026	0.954±0.029	0.8204
		Entropy	1.237±0.55	1.229±0.595	0.9665
	Level†: 10 Distance‡: 1 Orthogonal§	Contrast	0.117±0.064	0.121±0.069	0.8476
		Correlation	0.933±0.03	0.915±0.043	0.1458
		Uniformity	0.288(0.23–0.441)	0.337(0.22–0.412)	0.9621
		Homogeneity	0.942±0.031	0.94±0.034	0.833
		Entropy	1.518±0.573	1.508±0.607	0.9574
	Level†: 10 Distance‡: 3 Orthogonal§	Contrast	0.382±0.235	0.39±0.266	0.9203
		Correlation	0.792±0.086	0.747±0.117	0.1988
		Uniformity	0.227(0.153–0.376)	0.256(0.14–0.336)	0.9118
		Homogeneity	0.854±0.073	0.845±0.079	0.736
		Entropy	1.844±0.694	1.848±0.736	0.9861
	Level†: 10 Distance‡: 3 Diagonal§	Contrast	0.753(0.526–1.074)	0.707(0.411–1.103)	0.8619
		Correlation	0.55±0.135	0.481±0.144	0.1465
		Uniformity	0.179(0.121–0.33)	0.196(0.11–0.253)	0.937
		Homogeneity	0.774±0.088	0.759±0.097	0.6244
		Entropy	2.068±0.748	2.091±0.815	0.9282

*Std, standard deviation.

† Level, number of level.

‡ Distance, co-occurrence distance (pixel).

§ Orthogonal and diagonal, co-occurrence direction with ± (1,0) and ± (1,1), respectively.

**Table 3:** Comparison of textural parameters obtained from specimen photoacoustic images between tissues with psammoma bodies on pathology and those without when applying the mean value of the three slices of thyroid specimens.

		Paremeters	Mean ± SD or median (interquartile range)		*P* value
			Specimens without psammoma bodies	Specimens with psammoma bodies	
**Histogram**		Mean	40.375±17.453	35.283±14.927	0.352
		Std	26.386±14.789	20.917±11.579	0.2226
		Skewness	2.01±0.801	1.635±0.464	0.1027
		Kurtosis	11.063±6.403	7.986±3.199	0.0864
		Entropy	6.112±0.626	5.892±0.638	0.306
**Co-occurrence matrix**	Level†: 4 Distance‡: 1 Orthogonal§	Contrast	0.032(0.009–0.044)	0.015(0.005–0.055)	0.41
		Correlation	0.895(0.859–0.934)	0.857(0.839–0.91)	0.0527
		Uniformity	0.751±0.192	0.802±0.188	0.4263
		Homogeneity	0.984(0.978–0.996)	0.992(0.972–0.998)	0.41
		Entropy	0.529±0.389	0.414±0.372	0.3707
	Level†: 6 Distance‡: 1 Orthogonal§	Contrast	0.064±0.04	0.053±0.041	0.3991
		Correlation	0.903±0.041	0.896±0.038	0.6406
		Uniformity	0.559±0.227	0.629±0.237	0.3727
		Homogeneity	0.968±0.02	0.974±0.021	0.3984
		Entropy	0.956±0.51	0.79±0.51	0.3348
	Level†: 8 Distance‡: 1 Orthogonal§	Contrast	0.097±0.052	0.082±0.053	0.3857
		Correlation	0.918±0.033	0.907±0.035	0.3349
		Uniformity	0.42±0.202	0.486±0.213	0.3452
		Homogeneity	0.951±0.026	0.959±0.026	0.386
		Entropy	1.311±0.535	1.131±0.541	0.3243
	Level†: 10 Distance‡: 1 Orthogonal§	Contrast	0.129±0.062	0.109±0.061	0.3502
		Correlation	0.927±0.03	0.918±0.032	0.3584
		Uniformity	0.33±0.165	0.383±0.173	0.358
		Homogeneity	0.936±0.03	0.946±0.03	0.3583
		Entropy	1.594±0.544	1.412±0.538	0.3185
	Level†: 10 Distance‡: 3 Orthogonal§	Contrast	0.423±0.232	0.339±0.209	0.263
		Correlation	0.777±0.082	0.757±0.088	0.4741
		Uniformity	0.18(0.155–0.319)	0.288(0.146–0.458)	0.6122
		Homogeneity	0.84±0.071	0.86±0.071	0.3866
		Entropy	1.944±0.661	1.724±0.651	0.3223
	Level†: 10 Distance‡: 3 Diagonal§	Contrast	0.983±0.681	0.768±0.532	0.2963
		Correlation	0.532±0.092	0.491±0.099	0.2126
		Uniformity	0.214±0.159	0.262±0.171	0.3981
		Homogeneity	0.754±0.086	0.776±0.087	0.4435
		Entropy	2.185±0.728	1.956±0.717	0.3409

*Std, standard deviation.

† Level, number of level.

‡ Distance, co-occurrence distance (pixel).

§ Orthogonal and diagonal, co-occurrence direction with ± (1,0) and ± (1,1), respectively.

There were 8 patients who had higher mean values from samples with microcalcifications than those without microcalcifications on specimen PAIs (Figure 2). The remaining patients showed opposite results (Figure 3).

## Discussion

Microcalcifications within a thyroid nodule have been highly associated with thyroid cancer [17]–[19]. Therefore, several guidelines have accepted microcalcifications as a useful US feature in the diagnosis of thyroid cancer or in the selection of a thyroid nodule for fine needle aspiration [1], [20], [21]. However, there are two limitations in applying this US feature at a thyroid nodule in the clinical field. First, hyperechoic spots are not always related to microcalcifications. Second, assessing microcalcifications is subjective like the assessment of other US features. Reported interobserver variability shows a kappa value of 0.44–0.63 [22]–[25], which reflects the subjective nature of calcification assessment on US. Although US is the highest sensitive preoperative diagnostic tool for detecting a focal thyroid lesion, it does not show all microcalcifications which may be related to PBs [2]. Therefore, there is an increasing demand for a more sensitive and objective diagnostic tool in thyroid nodule assessment.

Photoacoustic imaging has been widely studied fused with variable medical fields such as US and microscopy [26], [27]. Recently, Kang et al. investigated PAI-visualized microcalcifications in breast tissues in a study which revealed the absorbance differences between microcalcifications and surrounding tissues [15], [28]. They demonstrated that a wavelength between 700 nm and 800 nm was the distinguishing absorption spectrum of microcalcifications in breast tissue [15]. Therefore, we investigated the diagnostic utility of PAI for thyroid microcalcifications at 700 nm laser wavelengths on the assumption that microcalcifications have the same absorption coefficients as breast tissue. In our study, we only included thyroid tissues with diffuse scattered punctate microcalcifications on specimen radiography and corresponding specimens without microcalcifications extracted from the same patient to minimize some factors such as underlying tissue changes which can impact the results of this study. We compared the parameters of texture analysis on PAIs in 3 different situations. First, we compared texture analysis obtained from the mean values of 3 specimen PAIs between tissues with punctate microcalcifications on specimen radiography and those without. As the mean value of histograms on US was higher in samples with microcalcifications on specimen radiography than those without, we can confirm that representative PAIs can show microcalcifications. However, the mean value of histograms on PAIs was lower in samples with microcalcifications on specimen radiography than ones without microcalcifications without statistical significance. Other parameters from texture analysis did not show any differences between them. Second, we selected the values of texture analysis from the slice of a thyroid specimen which had the highest value of the mean values in specimen US. However, there were no statistical significant parameters that differentiated samples with and without microcalcifications. Third, we compared textural parameters in specimens with PBs on pathology and those without because not all microcalcifications on specimen radiography correlate with PBs which can be related to a malignancy [2]. However, no significant differences were found between them as well.

From these results, it can be concluded that the textural analysis on PAI of thyroid microcalcifications with a linear array transducer does not indicate statistical significance in the spatial domain unlike that of breast microcalcifications. There can be several reasons that explain the different results between these similar studies on thyroid and breast tissues: blood coagulation on thyroid tissue samples may be the main artifact of our study because the thyroid gland is a highly vascular organ compared to breast tissue. From this structural difference between breast and thyroid tissue, we can assume that thyroid specimens may contain more blood that can coagulate between time durations for excision, resection and delivery to the imaging room for *ex vivo* experiments. Therefore, the relatively small endogenous PA signal from thyroid microcalcifications can be interfered with the oxy- and deoxy-hemoglobins which are the most significant light absorbers within the human body. In our study, the clustered hyperreactive PA signals were correspondingly shown on the reconstructed PA images. Those suspected to be from the residual blood coagulations are indicated by white arrows in Figures 2 and 3, respectively, despite the wash-out procedure with saline solution. Also, a technical issue arose in the scanning process for blood-free regions of the thyroid specimens. Although, a graduate researcher (K. J.) carefully selected slice positions to exclude blood coagulations, hyperreactive interference could not be completely excluded due to the broad slice thickness in the elevation direction of the US linear array transducer as simulated by Kang et al. (e.g., approximately 2 mm of slice thickness at 30-mm depth) [29]. Also, 3 dimensional images were not used in this study as microcalcifications on specimen radiography could not be correlated with US images. However, we assume that this limitation is negligible because the mean value of US images from samples with microcalcifications on specimen radiography was higher than that of US images from samples without microcalcifications.

In conclusion, PAI did not show significant difference in textural analysis on thyroid tissue with microcalcifications. This might be because PAI is more sensitive to tissue absorption changes rather than scattering changes, like microcalcifications, which can affect the result of our study. Thus, to be useful for clinical practice, the subtle light absorption change caused by microcalcifications should be differentiable in PA imaging. This requires an improvement in experimental setup and protocols and in turn, spatial resolution must be improved, especially in the elevation direction. For this, the 2-dimensional array transducer (e.g., xMATRIX transducer, Philips Electronics, Amsterdam, Netherlands) can be used which can provide narrower and uniformed elevation slice thickness throughout the imaging depth. Secondly, the complete blood rejection method for excised *ex vivo* specimens should be employed to solely evaluate the PA signals generated from thyroid microcalcifications. However, the ultimate solution should be the development of a multiwavelength spectroscopic PA imaging technique which can differentiate the microcalcifications from blood *in vivo* even in a highly interfered environment.
